# Patient vulnerability in stereotactic arrhythmia radioablation (STAR): a preliminary ethical appraisal from the STOPSTORM.eu consortium

**DOI:** 10.1007/s00066-024-02230-w

**Published:** 2024-04-23

**Authors:** Carlo Botrugno, Chiara Crico, Mauro Iori, Oliver Blanck, Slawomir Blamek, Pieter G. Postema, Aurelio Quesada, Etienne Pruvot, Joost J. C. Verhoeff, Ludovica De Panfilis

**Affiliations:** 1 Research Unit on Everyday Bioethics and Ethics of Science, Department of Legal Sciences, University of Florence, Florence, Italy; 2 Legal Medicine and Bioethics, Azienda USL-IRCCS di Reggio Emilia, Reggio Emilia, Italy; 3 Fondazione IRCCS Istituto Tumori, Milano, Italy; 4 Medical Physics Unit, Azienda USL-IRCCS di Reggio Emilia, Reggio Emilia, Italy; 5 Department of Radiation Oncology, University Medical Center Schleswig-Holstein, Kiel, Germany; 6 Department of Radiotherapy, Maria Skłodowska-Curie National Research Institute of Oncology, Gliwice, Poland; 7 Department of Clinical and Experimental Cardiology, Heart Failure & Arrhythmias, Amsterdam Heart Center and Cardiovascular Science, Amsterdam University Medical Centers, University of Amsterdam, Amsterdam, The Netherlands; 8 Cardiology Department, Arrhythmias Unit, Consorcio Hospital General Universitario de Valencia, Faculty of Medicine, Catholic University of Valencia “San Vicente Martir”, Valencia, Spain; 9 Heart and Vessel Department, Service of Cardiology, Lausanne University Hospital and University of Lausanne, Lausanne, Switzerland; 10 Department of Radiotherapy, University Medical Center Utrecht, Utrecht, The Netherlands; 11 Department of Radiotherapy, Amsterdam University Medical Centers, University of Amsterdam, Amsterdam, The Netherlands

**Keywords:** Ethics in research, Bioethics, Ventricular tachycardia, Cardiovascular disease, Stereotactic Body Radiotherapy

## Abstract

This preliminary ethical appraisal from the STOPSTORM.eu consortium is meant to raise critical points that clinicians administering stereotactic arrhythmia radioablation should consider to meet the highest standards in medical ethics and thus promote quality of life of patients recruited for radiotherapy treatments at a stage in which they experience a significant degree of vulnerability.

## Introduction

Cardiovascular disease is one of the leading causes of death in Europe (45%) [1]. In patients with structural heart disease, ventricular tachycardia (VT) is an unpredictable and potentially deadly condition, which is prevented by posing an implantable cardioverter defibrillator (ICD), antiarrhythmic medication and catheter ablation(s). When VT occurs, irreversible and potentially fatal organ damage may follow. Unfortunately, the combination of current treatments fails to prevent VT recurrence in 20–30% of VT patients [2, 3]. While patients can undergo multiple invasive ablations, they may refuse due to earlier complications, a (very) high chance of complications or perceived low efficacy; moreover, technical and clinical difficulties can lead to a lack of effective treatment options. A promising novel noninvasive treatment option for refractory VT is stereotactic arrhythmia radioablation (STAR) [4, 5]. STAR can be used to reach locations inaccessible to catheter ablation, which may potentially improve the effectiveness of overall VT treatment. Small-scale first trials in men and early-phase clinical trials have been performed for STAR, providing proof of concept for clinical safety and efficacy [6–8]. However, many questions remain, and the available studies lack the power to clinically validate the approach and prepare for phase III trials. To answer many of the remaining questions and to evaluate the efficacy and safety of STAR, a comprehensive database was needed. Therefore, the Standardized Treatment and Outcome Platform for Stereotactic Therapy of Re-Entrant Tachycardia by a Multidisciplinary (STOPSTORM.eu) consortium (Horizon 2020, GA no. 945119) consisting of 31 participating centres in eight European countries initiated a common database for both retrospectively and prospectively treated patients within the project duration [3]. The database is divided into an observational cohort (patients treated with STAR before or after the project started on 1 May 2021 but not according to STOPSTORM guidelines and patients treated following STOPSTORM guidelines but before the project started) and a prospective harmonized cohort (including patients treated prospectively under the guidelines defined during project runtime).

In this preliminary ethical appraisal, we raise some major points concerning the vulnerability of STAR patients by focusing our attention on the autonomy of choice, patients’ awareness about the implications of the treatment and “the last treatment option” issue. We aim to provide a valuable input to healthcare professionals performing STAR treatments to meet the highest standards in medical ethics, thus defending patients’ rights and promoting their quality of life.

## Materials and methods

The STOPSTORM project includes a dedicated work package aimed at drafting an ethical and legal framework for the administration of STAR. In such a context, priority will be given to assessing patients’ vulnerability when undertaking STAR and how their autonomy can be promoted in the clinical setting. The STOPSTORM project will then collect empirical evidence [9] on the ethical dimensions related to STAR treatments.

To pave the way for future empirical bioethics research with STAR patients, during the first 6 months of the STOPSTORM project, an interdisciplinary team worked on identifying the most relevant ethical issues and providing guidance that could be used by all consortium centres and associated project partners. International declarations and statements on human participation in research were coupled with notions and theories drawn from the field of bioethics and medical ethics and then framed into a preliminary assessment tailored to the main features and aims of the STOPSTORM project. Finally, the document containing a draft of the ethical and legal guidelines for the handling of the STOPSTORM project was shared and discussed with the ethics board members appointed for the project. The brief report presented herein draws from the content of these guidelines to focus on the vulnerability of patients who meet the conditions for STAR treatment [3].

## Defining vulnerability of patients in clinical research

The participation of patients in clinical research is a core issue in the field of medical ethics [10, 11] and has its main reference in the Declaration of Helsinki on the Ethical Principles for Medical Research Involving Human Subjects [12]. As known, special attention must be paid to the involvement of vulnerable participants, although there is no unanimous consensus on the definition of vulnerability in research, and multiple features may be stressed for assessing vulnerability of research participants [13, 14]. A definition provided by the US National Bioethics Advisory Commission in 2001 describes vulnerability in medical research as “a condition, either intrinsic or situational, of some individuals that puts them at greater risk of being used in ethically inappropriate ways in research” [15]. In the Declaration of Helsinki, vulnerability is described as “an increased likelihood of being wronged or of incurring additional harm” [12]. Accordingly, involving vulnerable patients in medical research is justified only “if the research is responsive to the health needs or priorities of this group and the research cannot be carried out in a non-vulnerable group” [12]. Therefore, recruited patients “should stand to benefit from the knowledge, practices or interventions that result from the research” [12]. The declaration also specifies that the ability to give consent is pivotal to assessing the vulnerability of research subjects: “Some research populations are particularly vulnerable and need special protection”, including those who cannot give or cannot refuse consent for themselves [12]. Another source for defining vulnerability in research comes from the Council for International Organizations of Medical Sciences [16], according to which “persons are vulnerable because they are relatively (or absolutely) incapable of protecting their own interests”. Not least, the Barcelona Declaration on Policy Proposals to the European Commission on Basic Ethical Principles in Bioethics and Biolaw [17] sees vulnerability as networked with “integrity, dignity and autonomy”. The intrinsic value of vulnerability would therefore come from it being not only the object of a moral principle, but for expressing the need for morality as it “requires not merely non-interference with the autonomy, dignity or integrity of beings, but also that they receive assistance to enable them to realize their potential” [17]. Besides the range of available definitions—which correspond to the huge debate on research vulnerability in bioethics and medical ethics—we consider the “contextual approach” (also known as “situational approach”) suitable to the needs of the STOPSTORM.eu consortium as it tries to overcome abstract categorizations and refers to “situations in which individuals might be considered vulnerable”. This kind of approach is based on contextual and relational evidence and the patient’s needs and it explicitly values connectivity, attentiveness and dialogue, participation, empowerment and critical reflexivity in the deployment of the doctor–patient relationship [18, 19]. From this perspective, the recognition of patients’ centrality is pivotal to dealing with the “contextual vulnerability” in an ethically compliant fashion. An example of the contextual approach is the so-called “shared decision-making” model, which is conceived to ensure that patients and clinicians can make decisions based on a mutual understanding of the case and after careful consideration of patients’ beliefs, preferences and attitudes [20].

## The vulnerability of patients undergoing STAR

Although the definition of vulnerability of research subjects may be highly variable, based on local practices, orientations and norms, the interdisciplinary teams working for the STOPSTORM.eu consortium have agreed on the fact that any patients suitable for STAR—regardless of whether they are eligible for clinical trial inclusion or access the treatment based on compassionate use—must be regarded as highly vulnerable. Such a view is not only because STAR is still a novel treatment and evidence of efficacy is still missing. The Declaration of Helsinki indeed allows physicians to use an unproven treatment on an individual if, in their judgment, they can offer “hope of saving life, re-establishing health or alleviating suffering” [12] and under the condition that “proven interventions do not exist or other known interventions have been ineffective” [12]. Despite the increasing opinion that STAR already represents a reasonable option for patients unresponsive to multiple standard treatments (both invasive and noninvasive treatments) [21], its administration unfolds some critical factors that deserve careful scrutiny from an ethical point of view.

First, STAR takes cardiology patients into treatment by radiation oncologists guided by cardiologists who define the target region to be treated by radiation. This transition should not be underestimated as it creates a new scenario in most centres, one in which critical issues can arise in clinical practice, especially if coordination between professionals from different specialties involved in STAR is not practised and seamless. In other words, as a complex and novel treatment, the administration of STAR calls for a “professional alliance” that enables cardiologists and radiation oncologists to establish a well-rehearsed and well-defined cooperation and sharing of information—and not least, responsibility. This is meant to avoid a detrimental impact on the singularity of each case, and therefore to ensure protection of suitable patients as research participants.

Secondly, STAR is a treatment that is targeted to critically ill patients. This entails that research participants possibly find themselves in a final stage of their life, which can significantly restrict their autonomy of choice when it comes to adhering to a STAR research protocol. Particularly challenging for this group of patients can be developing a sufficient awareness of the treatment’s risks and their expected benefits, i.e., how and whether they are well balanced in their cases. The patients’ lack of awareness about the experimental treatment they undergo may lead to therapeutic misconception and/or to the difficulty of participants to “distinguish between research and clinical care” [22]. As unambiguously emphasized by the Declaration of Helsinki in this regard [12], informed consent is pivotal to protecting patients and respecting their dignity and autonomy of choice. Nevertheless, the history of medicine includes plenty of episodes where patients suitable for unproven treatments have been “nudged” to encourage their recruitment in a study marketed to be the “best option” for them.

Such a scenario can be facilitated by the asymmetric power relationships between patients as research subjects and clinicians as researchers. Evidence indeed shows that research participants seldomly develop a proper understanding of the features of the clinical experimentation to which they adhere [22]. Moreover, a positive correlation between (higher) levels of education and understanding of informed consent forms has been reported [22], confirming that these forms do not always meet the levels of literacy of the general population. To stimulate full awareness of the implications of STAR, i.e., to provide patients with an effective understanding of the features, purposes and limitations of this novel treatment, the STOPSTORM.eu consortium has already drafted ethical guidelines for creating informed consent forms that support comprehension by avoiding technical jargon and prioritizing short and simple sentences. In parallel to this, the request for consent to recruitment has been unambiguously distinguished from the request for personal data collection.

A third factor relevant to the vulnerability of STAR patients is that the latter may be unable to express their consent to the novel treatment due to their clinical conditions (e.g., considering the case of patients in coma due to ventricular storm [24–26]). The Clinical Trials Regulation (EU) no. 536/2014 (Art. 31) specifies that in the case of “incapacitated subjects, who have not given, or have not refused to give, informed consent before the onset of their incapacity”, a clinical trial may be conducted only if some special conditions are met [23]. These include obtainment of informed consent from their legally designated representative and the absence of any incentives or financial inducements for the research participants or for their legally designated representatives. The trial must also be “essential with respect to incapacitated subjects and data of comparable validity cannot be obtained in clinical trials on persons able to give informed consent, or by other research methods”. Furthermore, the trial relates to a medical condition from which the participants suffers and there are scientific grounds for expecting “a direct benefit to the incapacitated subject outweighing the risks and burdens involved” from the participation in the research. National provisions provide more specific (possibly severe) rules to deal with the case of incapacitated patients and the impossibility of obtaining consent from them, possibly leading to notable differences from country to country.

Beside the case of a total lack of capacity, there can be a huge variety of situations in which the ability to give consent can be only partial. In this regard, as also highlighted by the Declaration of Helsinki, if a potential participant assumed to be unable to give consent is anyhow able to give it, “the physician must seek that assent in addition to the consent of the legally authorised representative” [12]. The evaluation of patients’ capacity to give consent assumes a pivotal role in the administration of STAR, conferring a special responsibility to the clinicians involved.

Here, it is worth reminding that the impossibility to give consent is not a “mere” legal issue, but also involves a moral question. Professionals administering STAR therefore have a special responsibility in this situation, as they can use their relational skills and experience to handle the lack of patient autonomy to elicit valuable information from family members and a patient’s legally designated representatives, with the ultimate purpose of making a decision that can be seen as respectful of the concerned patients.

## Conclusion

STAR represents a promising novel noninvasive treatment option for refractory VT. The STOPSTORM.eu consortium primarily aims at collecting evidence for its efficacy and safety by building a common database for patients treated with STAR. Nevertheless, it also seeks to guide consortium centres—as well as any other centres administering STAR—to comply with the highest standards in medical ethics, thus safeguarding patients’ autonomy and protecting their rights. The future empirical research foreseen under the ethics work program of the STOPSTORM.eu consortium will generate evidence related to the subjective experience of patients enrolled in STAR trials, disclosing further insights into patients’ vulnerability and paving the way toward the drafting of evidence-based ethical guidelines for handling it. In the meantime, this preliminary ethical appraisal is meant to raise the attention of the community of clinicians involved in STAR treatment regarding some critical points that deserve careful scrutiny from an ethical point of view. In particular, we focused on three factors from which the specific condition of vulnerability of STAR patients may depend: the novelty of cardiology patients being treated by radiation oncologists, the risk that patients may be subject to pressure to adhere to experimental research protocols, and the total or partial lack of patients’ ability to give consent seen as a moral question rather than solely a legal issue. We hope that this appraisal can be a reference for all clinicians committed to STAR investigations in routine care to combine the novelty of the treatment with the most consolidated safeguards established by medical ethics in defence of patients’ rights, dignity and autonomy.

## Data Availability

Not applicable as research data are not included in this manuscript.
